# Optimization Scheme for 3D Printing of PLA–PHBV–PCL Biodegradable Blends for Use in Orthopedic Casting

**DOI:** 10.3390/polym17070852

**Published:** 2025-03-22

**Authors:** Muhammad Mohid Aziz, Logan Beard, Shafahat Ali, Abdelkrem Eltaggaz, Ibrahim Deiab

**Affiliations:** 1 Advanced Manufacturing Lab (AML), School of Engineering, University of Guelph, Guelph, ON N1G 2W1, Canada; 2 Mechanical Engineering Department, Australian University of Kuwait, Kuwait City 13015, Kuwait

**Keywords:** optimization, 3D printing, bioplastics, orthopedic casting

## Abstract

Three-dimensional printing technology offers significant advantages in the production of orthopedic casts, providing a promising alternative to conventional plaster and fiberglass materials. Polylactic acid (PLA) is widely used for this purpose; however, its adoption is limited due to poor mechanical properties, including high brittleness, low thermal stability, and limited elongation. These challenges can be mitigated by blending PLA with other biodegradable polymers. This study investigated a blend of PLA with poly(3-hydroxybutyrate-co-3-hydroxyvalerate) (PHBV), a type of polyhydroxyalkanoate (PHA), and polycaprolactone (PCL) for the development of 3D printed orthopedic casts. The key mechanical properties—tensile strength, percent elongation at break, Young’s modulus, flexural strength, flexural modulus, and impact strength—were evaluated as a function of the printing parameters, including nozzle temperature, layer height, and raster angle. The grey relational analysis (GRA) approach was applied to optimize these mechanical properties. The optimal printing parameters were found to be a nozzle temperature of 180 °C, a layer height of 0.18 mm, and a raster angle of 0°, resulting in a tensile strength of 44.4 ± 4.4 MPa, an elongation at break of 68.5 ± 11.6%, a Young’s modulus of 948.7 ± 25.1 MPa, a flexural strength of 54.6 ± 8.9 MPa, a flexural modulus of 1549.3 ± 141 MPa, and an impact strength of 80.77 ± 5.6 J/m. Statistical analysis using analysis of variance (ANOVA) revealed that for tensile strength, 50.18% was influenced by the raster angle, 26.38% by the layer height, and 18.92% by the nozzle temperature; for flexural strength, 69.81% was influenced by the raster angle, 20.67% by the layer height, and 3.53% by the nozzle temperature; and for impact strength, 75.11% was influenced by the raster angle, 13.16% by the layer height, and 4.45% by the nozzle temperature.

## 1. Introduction

Twenty-four out of every thousand individuals suffer from one or more bone fractures in their lives [1]. Orthopedic casts, used to heal these fractures, have been traditionally made out of plaster or fiberglass [1]. However, these casts have several issues such as potential skin disease development, poor ventilation, discomfort, and pressure-related complications [1,2]. Three-dimensional printing technology can be used to produce orthopedic casts, which provides several advantages over conventional plaster and fiberglass casts, such as reducing the number of physician visits required, improved function, less skin problems, being waterproof and washable, being self-removable, and a reduction in pressure-related complications [2,3].

Three-dimensional printing is a rapidly advancing technology and has a wide range of applications in different fields, such as transportation, healthcare, and aerospace [4,5], automotives, biomedical applications [6,7], electronics [8], packaging, and construction [9,10,11,12]. Among these materials, polylactic acid (PLA) has become a popular choice for 3D printing due to its favorable properties, including a low melting temperature [13], high strength [14], low cost, lightweight structure [15], an ease of printing [16], and biodegradability [17,18,19]. These characteristics make PLA especially suitable for medical applications, where the demand for sustainable materials is growing. PLA’s biodegradability is particularly attractive considering the plastic waste crisis, with over 6.3 billion metric tons of plastic contaminating natural environments worldwide [20,21,22,23]. However, while PLA shows promise, its use is limited in certain applications due to its high brittleness, low thermal stability [24], and restricted flexibility, which can reduce its suitability for more rigorous medical applications [14].

To overcome these challenges, researchers have investigated blending PLA with other biodegradable polymers to improve its mechanical properties, resulting in stronger and more flexible materials. Studies have demonstrated that blending PLA with polymers like polycaprolactone (PCL) and polyhydroxyalkanoates (PHA) can significantly enhance its mechanical properties, including toughness and elongation at break. PLA can be blended with other biodegradable polymers to increase its mechanical properties according to specific requirements. Qiu et al. [25] found an increasing toughness and elongation at break with an increasing PCL content in a PLA/PCL blend ranging from 0–40% PCL. Burzic et al. [26] found that PHAs increase the impact resistance of PLA. Su et al. [27] demonstrated a 400% increase in toughness by adding less than 1% of benzoyl peroxide (BPO) and dicumyl peroxide (DCP) into a blend of PLA and polybutylene succinate (PBS). Additionally, a blend of 35% PLA and 65% polybutylene adipate terephthalate (PBAT) resulted in a break elongation of approximately 304% compared to pure PLA [28]. Polymer blends can be created by fused deposition modeling (FDM), which enables multiple materials to be deposited simultaneously in a layer-by-layer manner [29,30].

The mechanical properties of 3D printed parts produced using FDM are influenced by various printing parameters, such as raster angle, layer thickness, and nozzle temperature. Research has shown that these parameters can have a significant impact on the mechanical performance of printed parts, affecting properties such as tensile strength, elongation, and impact resistance [31,32,33]. Ding et al. [34] investigated the effect of nozzle temperature on mechanical properties and found that nozzle temperatures ranging from 380–420 °C had varied effects on different mechanical properties. Rankouhi et al. [33] investigated the effect of layer thickness on mechanical properties and found that a 0.2 mm thick layer showed better properties than a 0.4 mm layer. Es-Said et al. [35] investigated the effect of raster orientation on tensile, flexural, and impact properties and found an angle of 0° to be the strongest in all three cases.

The current study introduces a novel approach by exploring the use of a ternary PLA blend with polyhydroxyalkanoates (PHBV) and polycaprolactone (PCL) for 3D printed orthopedic casts. Traditional orthopedic materials such as plaster and fiberglass often exhibit limitations, including brittleness, poor thermal stability, and restricted flexibility, which can compromise their performance in medical applications. Previous studies have focused on improving PLA’s mechanical properties by blending it with other biodegradable polymers. For instance, Qiu et al. [25] demonstrated that blending PLA with PCL improves its toughness and elongation at break. Similarly, Burzic et al. [26] found that incorporating polyhydroxyalkanoates (PHAs) into PLA increased its impact strength, providing more robust materials for medical use. These findings are intended to enhance PLA’s performance by combining it with PHBV and PCL in this study. What distinguishes the current investigation from previous studies is its comprehensive analysis of multiple mechanical properties, including tensile strength, the elongation at break, Young’s modulus, flexural strength, flexural modulus, and impact strength, to determine the optimal blend for orthopedic applications. Unlike earlier works, this research simultaneously addresses the combined effects of critical 3D printing parameters—layer thickness, raster angle, and nozzle temperature—on the mechanical properties of the PLA–PHBV–PCL blend. While Ding et al. [34] and Es-Said et al. [35] explored the effect of individual printing parameters on PLA-based materials, this study integrates these parameters into a multi-response optimization approach using grey relational analysis (GRA), a technique that allows for the simultaneous optimization of several factors. This multi-faceted approach offers a more holistic understanding of how 3D printing variables impact the final product’s mechanical properties, thereby providing a more effective strategy for developing high-performance materials for 3D printed orthopedic casts. Moreover, the use of GRA for optimizing printing parameters in combination with biodegradable materials such as PLA, PCL, and PHBV makes this study particularly innovative. The proposed approach promises to significantly improve the mechanical properties of orthopedic casts, ensuring they are more durable, flexible, and suitable for clinical use. Additionally, the integration of biodegradable materials contributes to addressing the growing concern over plastic waste in medical applications, making this study not only a technical advancement but also a step forward in terms of environmental sustainability.

## 2. Materials and Methods

### 2.1. Materials

**Figure 1:**
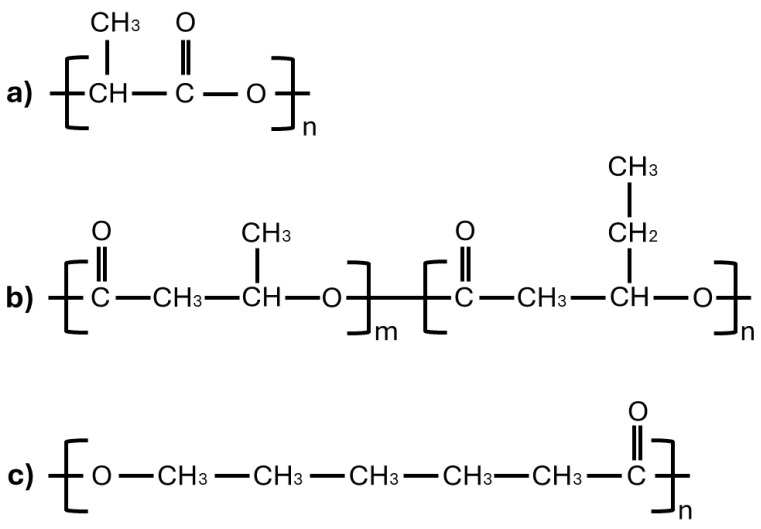
Chemical structures of (**a**) PLA, (**b**) PHBV, and (**c**) PCL.

Ingeo 4043D PLA pellets from NatureWorks LLC (Plymouth, MN, USA), Enmat Y1000P PHBV pellets from TianAn Biologic Materials(Ningbo, China), and Polycaprolactone MW 25000 PCL pellets from Polysciences Inc. (Warrington, PA, USA). The composition of the filament was selected to be 70% PLA, 20% PHBV, and 10% PCL and their molecular structure has been shown in Figure 1.

### 2.2. Filament Extrusion

**Figure 2:**
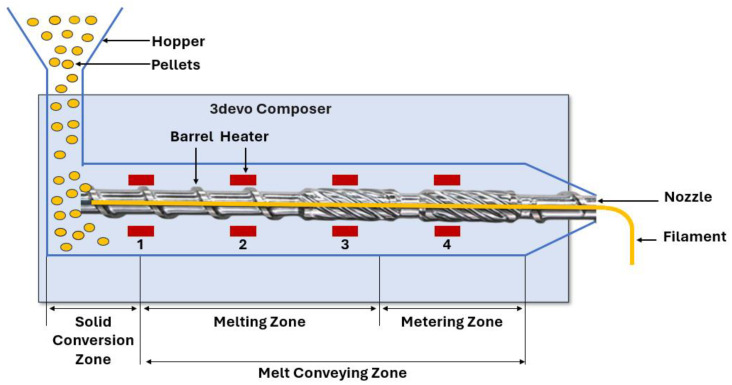
Diagram of 3devo filament extruder.

A filament of 1.75 mm diameter was extruded from the pellets using a 3devo Composer 350, a double screw extruder. The filament extruder was equipped with a built-in monitoring system which keeps the diameter of the filament at 1.75 ± 0.15 mm. Heat zones 1–4 of the extruder were set at 165 °C, 170 °C, 180 °C, and 170 °C, respectively, according to the manufacturer’s recommendations shown in Figure 2. Heat zone 1 was kept lower than the others to avoid PCL from sticking to the other polymers before melting. After extrusion, the filament was dried in an oven at 50 °C for 24 h.

### 2.3. Design of Experiment

**Figure 3:**
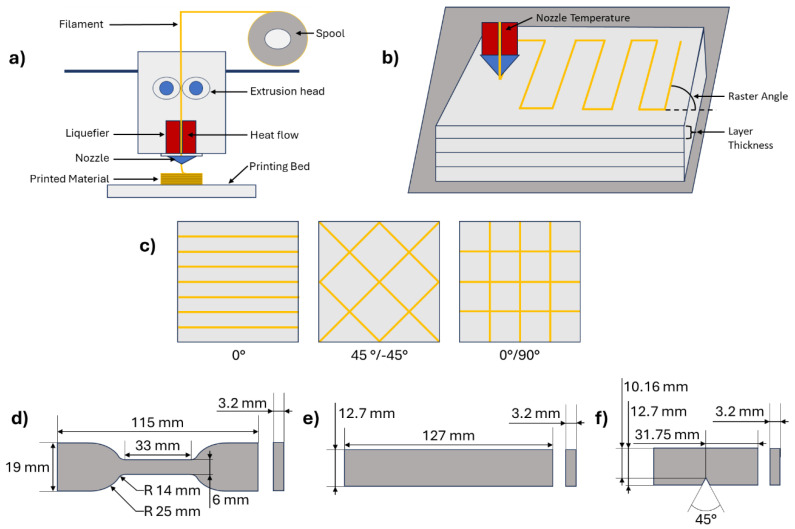
Schematic graphics of the (**a**) 3D printer, (**b**) printing parameters, (**c**) raster angles, (**d**) tensile sample, (**e**) flexural sample, and (**f**) impact sample.

The Bambu X1 Carbon Series 3D printer was used for printing samples. The constant parameters used include a 50 °C bed temperature, 100% infill density, 0.60 mm nozzle diameter, and a rectilinear printing pattern. Varied parameters consisted of the nozzle temperature, the layer height, and the raster angle as shown in Figure 3. The nozzle temperature varied between 180 °C, 190 °C, and 200 °C based on the pre-set of experiments. Temperatures below 180 °C resulted in improper material extrusion, and temperatures above 200 °C caused material to drip from the nozzle. The layer height varied between 0.18 mm, 0.3 mm, and 0.42 mm. The layer thickness selection followed the rule that it should be from 25 to 75% of the nozzle diameter [36,37]. As the nozzle diameter is 0.6 mm, the values selected were required to be between 0.15 mm and 0.45 mm. The raster angle varied between 0, ±45, and 0/90 degrees.

A Taguchi L9 experimental design was used to investigate the influence of various printing parameters. The design of the experiment is outlined in Table 1. This method allows for the efficient evaluation of multiple factors and their interactions with a minimal number of experiments [12,38,39].

### 2.4. Mechanical Testing

The tensile and flexural testing was performed on the Instron Universal Testing Machine (Figure S1). A load of 5 kN with a crosshead speed of 5 mm/min using the ASTM standard D638 was used for tensile testing, and a load of 5 kN with a crosshead speed of 14 mm/min using the ASTM standard D790 was used for flexural testing [40,41]. Izod pendulum impact testing was performed on a pendulum testing machine (Figure S1) using the ASTM standard D256 [42].

### 2.5. Scanning Electron Microscope

An Inspect FE50 scanning electron microscope (SEM) was used to explore the morphology of the PLA/PHBV/PCL blends. An accelerating voltage of 20 kV and a field emission gun were used. Samples were observed perpendicular to their fractured surfaces.

## 3. Results and Discussion

### 3.1. Effect of Parameters on Tensile Properties

**Figure 4:**
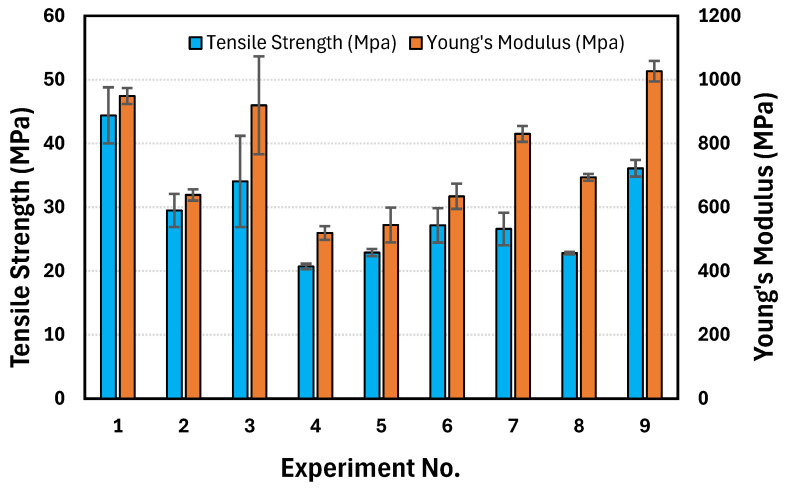
Graphical results of tensile strength and Young’s modulus.

**Figure 5:**
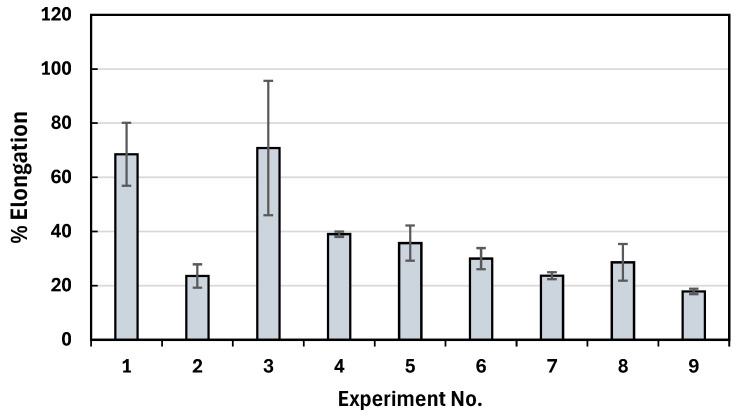
Graphical results of percent elongation at break.

Figure 4 and Figure 5 illustrate the impact of different printing parameters on the tensile properties of PLA/PHBV/PCL blends. The mechanical performance of the printed samples was highly dependent on process parameters such as nozzle temperature, layer height, and raster angle, which influence the degree of fusion between the extruded layers, internal porosity, and molecular orientation. As shown in Figure 4 and Table S1, Experiment No. 1 (180, 0.18, 0) resulted in the highest tensile strength of 44.4 ± 4.4 MPa. This may be attributed to the optimized balance between molecular diffusion and interlayer bonding at 180 °C, which facilitates adequate polymer chain entanglement without excessive degradation. The 0.18 mm layer height promoted better adhesion between layers, reducing defects and voids within the structure. Additionally, the 0° raster angle (aligned parallel to the loading direction) ensured that the tensile force is applied along the continuous filament deposition path, minimizing stress concentrations and enhancing load transfer efficiency. In contrast, as shown in Figure 5 and Table S1, Experiment No. 3 (200, 0.42, 0) resulted in the highest percentage elongation at break (70.8 ± 24.8%), which was a significant increase compared to PLA which has 2–5% elongation at break. The increased nozzle temperature of 200 °C enhanced polymer chain mobility and interlayer diffusion, while the larger 0.42 mm layer height introduced increased flexibility, contributing to higher strain tolerance before failure. Additionally, as shown in Figure 4 and Table S1, Experiment No. 9 (200, 0.18, 0/90) resulted in the highest Young’s modulus at 1026.45 MPa. The 0/90° raster angle introduced a cross-hatched pattern, increasing the stiffness and resistance to deformation due to multi-directional load distribution. The 200 °C temperature further ensured enhanced interlayer adhesion, contributing to the improved modulus.

**Figure 6:**
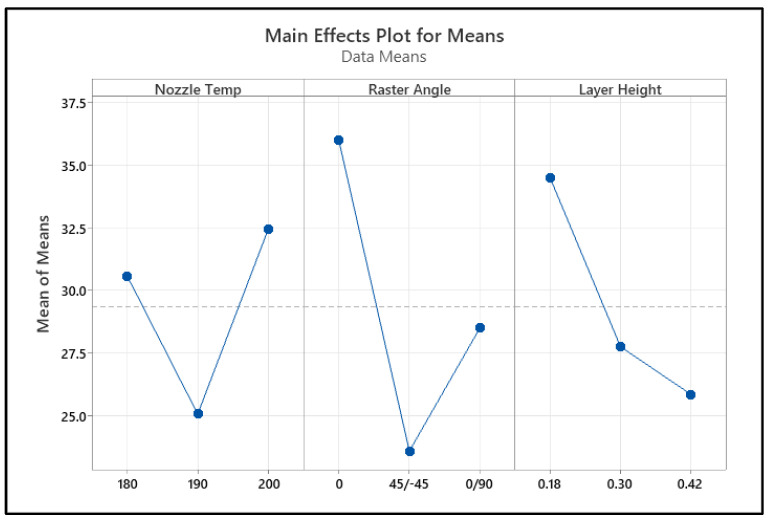
Main effect of the process parameters on tensile strength.

The decrease in tensile strength with an increase in raster angle can be attributed to the alignment of printed filaments relative to the applied load. When the raster angle is 0°, the printed fibers are fully aligned with the tensile loading direction, allowing for the efficient transfer of stress along the length of the filaments, resulting in higher tensile strength. However, as the raster angle increases (e.g., 45° or 90°), the load is no longer directly transferred along the filament direction but is instead distributed across multiple filament orientations. This misalignment increases the stress concentration at the interlayer regions, leading to weaker bonding and a higher likelihood of premature failure at the layer interfaces [43]. Additionally, at higher raster angles (e.g., 90°), the tensile force primarily acts perpendicular to the filament deposition, where the adhesion between layers (rather than the intrinsic filament strength) becomes the dominant load-bearing mechanism. Since interlayer bonding is generally weaker than intralayer bonding, the overall tensile strength is reduced. Tensile strength decreased with an increase in layer height. Higher layer heights lead to the formation of voids, reducing the tensile strength of the structure of the material [43]. A main effect plot was constructed using the mean value of each parameter at each variable level. The highest valued variable levels determined the optimal combination of the parameters. As shown in Figure 6, the optimal performance for tensile strength can be obtained at a raster angle of 0°, a layer height of 0.18, and a nozzle temperature of 180 °C.

### 3.2. Effect of Parameters on Flexural Properties

**Figure 7:**
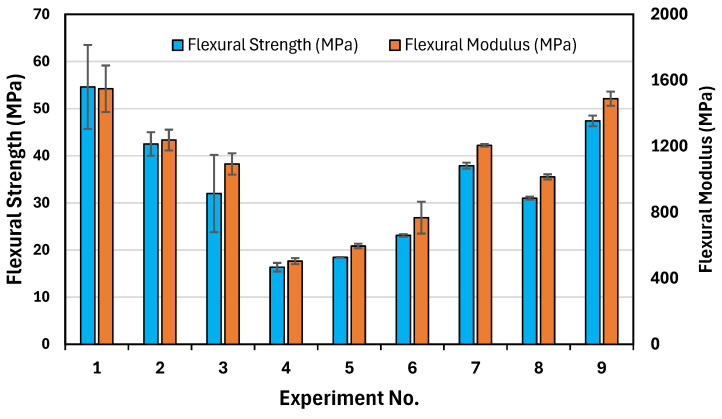
Graphical results of flexural strength and flexural modulus.

Figure 7 illustrates the impact of different printing parameters on the flexural properties of PLA/PHBV/PCL. As shown in Figure 7 and Table S1, the highest flexural strength of 54.6 MPa was achieved with Experiment No. 1 (180, 0.18, 0). Similarly, the highest flexural modulus of 1549.3 MPa was also recorded under the same conditions. Comparing tensile and flexural properties, a similar trend was observed—both tensile and flexural strength were highest at a 0° raster angle, where the filaments were aligned with the loading direction, ensuring efficient stress transfer. However, tensile strength decreased more significantly with an increasing raster angle compared to flexural strength, likely due to the different stress distributions in the tensile and bending loads. In flexural testing, the outermost layers experienced the highest stress, and the presence of compressive and tensile forces within the material may reduce the dependency on raster orientation compared to pure tensile loading. Additionally, the flexural modulus followed a trend similar to the Young’s modulus, where the higher raster angles (e.g., 90°) resulted in lower modulus values due to weaker interlayer bonding. The correlation between the tensile and flexural properties suggests that similar optimization strategies, such as fine-tuning the nozzle temperature and layer height, could enhance both properties for improved mechanical performance.

**Figure 8:**
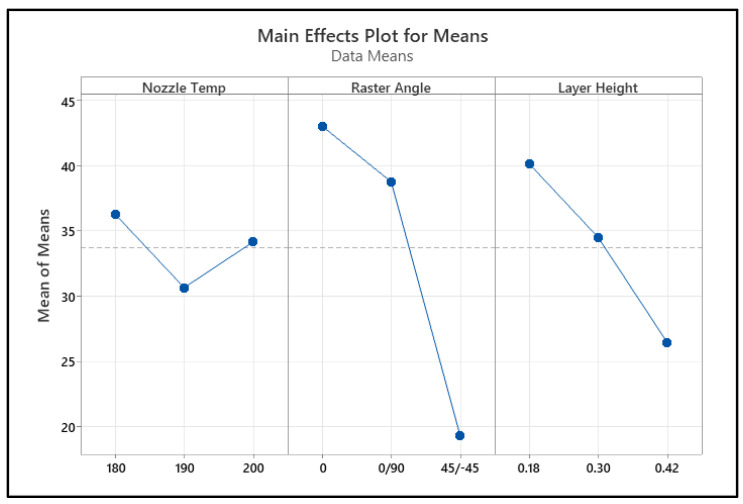
Main effect of process parameters on flexural strength.

Flexural strength decreased with an increase in raster angle. Lower angles exhibited stronger interlayer bonding compared to higher raster angles, and the deposited layers were closer to parallel to the bending plane, which offers more resistance to the bending force [44]. Flexural strength decreased with an increase in layer height. At lower layer heights, stronger bonding occurred between the layers, resulting in higher flexural strength [44]. A main effect plot was constructed using the mean value of each parameter at each variable level. The highest valued variable levels determined the optimal combination of the parameters. As shown in Figure 8, the optimal performance for flexural strength can be obtained at a raster angle of 0°, a layer height of 0.18 mm, and a nozzle temperature of 180 °C.

### 3.3. Effect of Parameters on Impact Properties

**Figure 9:**
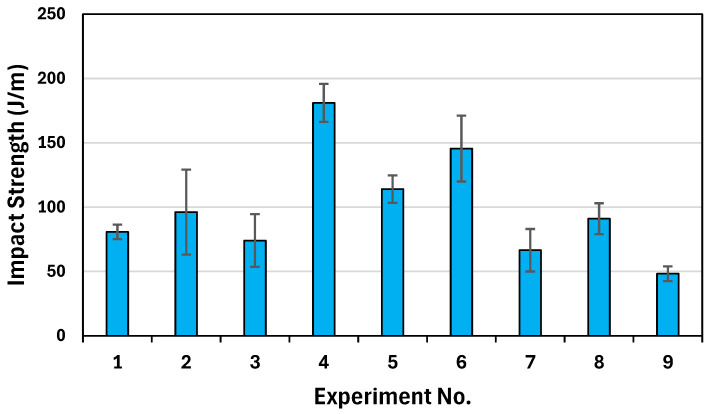
Graphical results of impact strength.

Figure 8 illustrates the impact strength of the PLA/PHBV/PCL composites under different printing conditions. As shown in Figure 9 and Table S1, the highest impact strength of 181.04 J/m was achieved in Experiment No. 4 (180, 0.42, 45).

The increase in impact strength with increasing layer height can be attributed to enhanced energy absorption capacity. At higher layer heights, the material contains a greater volume per layer, allowing for more effective dissipation of the impact energy through deformation and fracture mechanics. In contrast, lower layer heights create more densely packed layers with fewer voids, which, while beneficial for tensile and flexural strength, may reduce the material’s ability to absorb impact forces effectively [43].

The increase in impact strength with raster angle is primarily due to improved filament interlocking and multidirectional stress distribution. At higher raster angles (e.g., ±45°), the deposited filaments cross over one another, enhancing the interlayer adhesion and internal reinforcement. This arrangement allows the structure to distribute impact forces across multiple directions, preventing localized stress concentration and enabling greater energy absorption before fracture. The combination of shear failure and energy dissipation through interlayer deformation results in higher impact strength at greater raster angles. Conversely, the decrease in impact strength with increasing nozzle temperature is likely due to reduced void content. While a higher nozzle temperature improves layer bonding and reduces porosity, it also reduces the ability of micro voids to absorb impact energy. At lower temperatures, small voids may form at the interlayer interfaces, which can act as micro-energy absorbers under impact loading. However, at excessively high nozzle temperatures, the increased flowability of the material can lead to denser structures with fewer voids, resulting in brittle behavior and lower impact strength [37,43].

**Figure 10:**
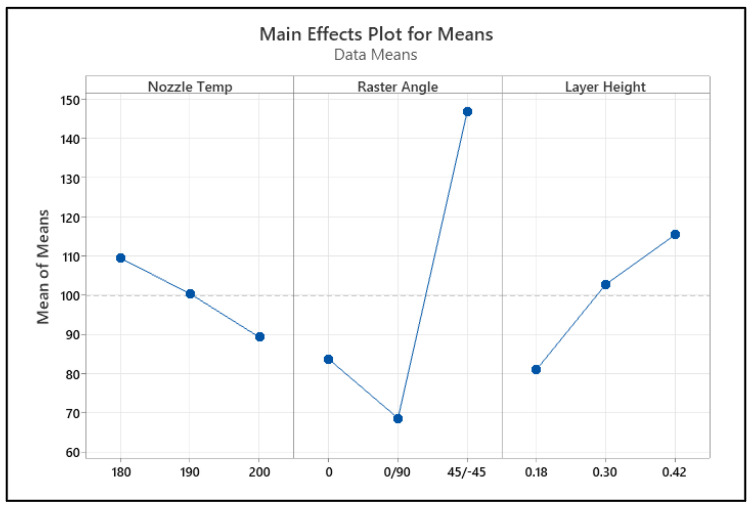
Main effect of process parameters on impact strength.

A main effect plot was constructed using the mean value of each parameter at each variable level. The highest valued variable levels determined the optimal combination of the parameters. As shown in Figure 10, the optimal performance for impact strength can be obtained at a raster angle of ±45°, a layer height of 0.42 mm, and a nozzle temperature of 180 °C.

### 3.4. Surface Morphology Analysis

**Figure 11:**
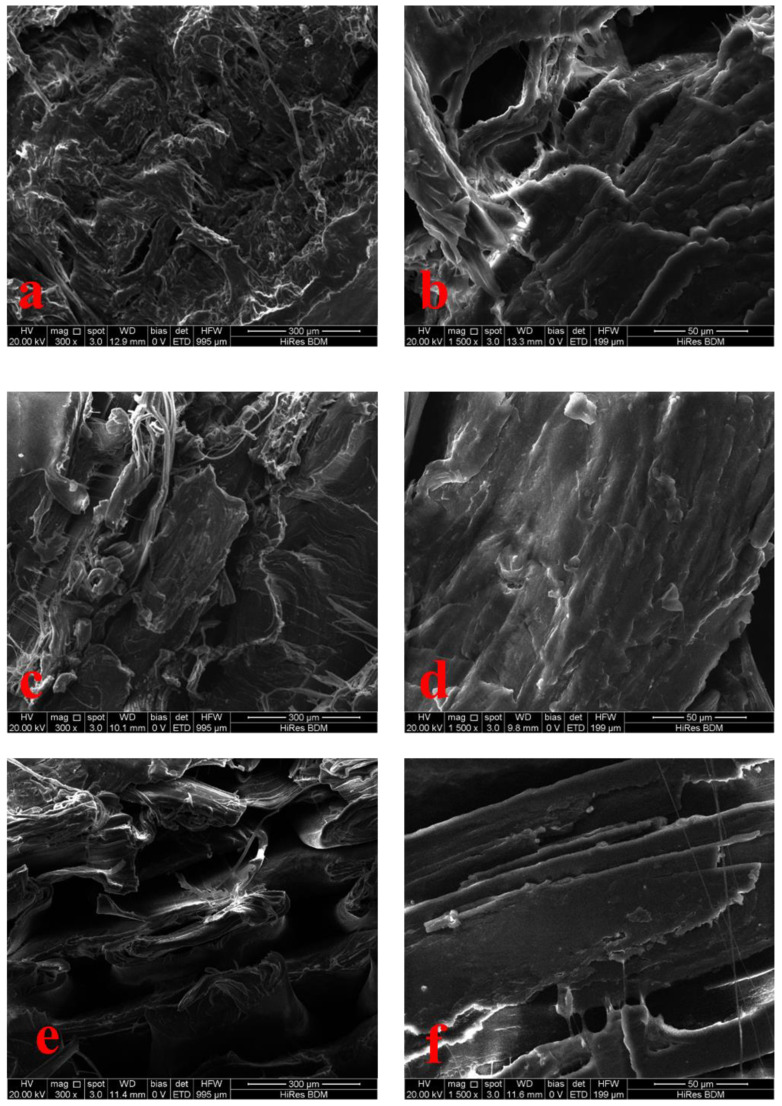
Morphology of impact-fractured specimens under varying printing conditions for (**a**,**b**) Experiment No. 4, (**c**,**d**) Experiment No. 8, and (**e**,**f**) Experiment No. 9.

The SEM images presented in Figure 11a,b for Experiment No. 4 revealed strong interlayer adhesion, which is crucial for maintaining the material’s structural integrity. The clear, cohesive bonding between the printed layers suggests that the optimized printing parameters, such as The nozzle temperature, raster angle, and layer height, were effectively controlled, leading to solid layer bonding without visible gaps. This is important because such strong interlayer adhesion contributes to the material’s overall mechanical strength and impact resistance. On the other hand, Figure 11e,f, representing Experiment No. 11, showed noticeable gaps and poor bonding between the layers. These visible separations indicate that the printing parameters used in this experiment were suboptimal, likely due to insufficient temperature or improper extrusion settings, which resulted in a significant reduction in interlayer adhesion. This lack of bonding weakens the material, making it more prone to failure under stress, as seen in its diminished impact properties. Similarly, in Experiment No. 8 (Figure 11c,d), the SEM images demonstrated partial interlayer adhesion, but there were still visible flaws such as small gaps or incomplete bonding between the layers. While the material exhibited some degree of adhesion, these inconsistencies contributed to lower impact resistance and mechanical performance.

### 3.5. Optimization of Printing Parameters

The grey relation analysis (GRA) approach was used to make multi-criteria conclusions. The GRA ranks the optimized data to identify the optimal settings. The grey relational coefficient was determined between all comparability sequences and the reference sequence, which was used to determine the grey relational grades, and these values are illustrated in Table 2.

The significance of each parameter on tensile strength (Table S2), flexural strength (Table S3), and impact strength (Table S4) was obtained by analyzing the observations through analysis of variance (ANOVA) [25]. It found that, for tensile strength, the percent contribution of each factor was 50.18% for the raster angle, 26.38% for the layer height, and 18.92% for the nozzle temperature. For flexural strength, the percentage contribution of each factor was 69.81% for the raster angle, 20.67% for the layer height, and 3.53% for the nozzle temperature. For impact strength, the % contribution of each factor was 75.11% for the raster angle, 13.16% for the layer height, and 4.45% for the nozzle temperature.

The analysis demonstrated that temperature, speed, and layer height significantly impacted the crystallinity and mechanical properties of the recycled PHA. Specifically, increasing the temperature resulted in decreased crystallinity but enhanced mechanical properties. This study investigated the optimization of 3D printing parameters to enhance the quality and mechanical properties of parts made from recycled plastics. Key parameters included extrusion temperature, nozzle diameter, raster angle, nozzle temperature, orientation, infill ratio, layer height, printing speed, and nozzle size. The findings indicated that these parameters significantly impact the mechanical performance and dimensional accuracy of the 3D printed parts. Properly adjusting these parameters can improve tensile strength, bending strength, ductility, and impact strength resistance. However, they must be carefully balanced to avoid issues like filament decomposition or reduced dimensional tolerances.

## 4. Conclusions

This study explored the 3D printing of a biodegradable blend composed of PLA, PHBV, and PCL. The mechanical properties of the printed samples were optimized by varying the following printing parameters: nozzle temperature, layer thickness, and raster angle. The key findings included the identification of optimal printing conditions (180 °C nozzle temperature, 0.18 mm layer height, and 0° raster angle), resulting in 3D printed samples with superior tensile strength, percent elongation at break, Young’s modulus, flexural strength, flexural modulus, and impact strength. The mechanical properties obtained with these parameters had a tensile strength of 44.4 ± 4.4 MPa, a percent elongation of 68.5 ± 11.6%, a Young’s modulus of 948.7 ± 25.1, a flexural strength of 54.6 ± 8.9 MPa, and an impact strength of 80.77 ± 5.6.

A 0° raster angle had higher tensile and flexural properties, as samples made under this condition rely less on interlayer bonding to maintain integrity, which is inherently weak. However, a ± 45° angle had the highest impact strength. A smaller layer height was better for tensile and flexural properties, and a larger layer height was better for impact strength. Larger layers tended to have more voids, which caused lower tensile and flexural properties, but added impact strength.

It was found that tensile strength was influenced 50.18% by the raster angle, 26.38% by the layer height, and 18.92% by the nozzle temperature; flexural strength was influenced 69.81% by the raster angle, 20.67% by the layer height, and 3.53% by the nozzle temperature; and impact strength was influenced 75.11% by the raster angle, 13.16% by the layer height, and 4.45% by the nozzle temperature.

## Figures and Tables

**Table 1:** Design of Taguchi L9 experiment.

Experiment No.	Nozzle Temperature (°C)	Layer Height (mm)	Raster Angle (deg)
1	180	0.18	0
2	190	0.3	0
3	200	0.42	0
4	180	0.42	±45
5	190	0.18	±45
6	200	0.3	±45
7	180	0.3	0/90
8	190	0.42	0/90
9	200	0.18	0/90

**Table 2:** GRA rank table to find optimal properties.

Experiment No.	Tensile Strength	Percent Elongation at Break	Young’s Modulus	Flexural Strength	Flexural Modulus	Impact Strength	Grade	Rank
1	1	0.92	0.77	1	1	0.4	0.85	1
2	0.44	0.36	0.4	0.61	0.63	0.44	0.48	4
3	0.53	1	0.7	0.46	0.53	0.38	0.6	3
4	0.33	0.45	0.33	0.33	0.33	1	0.46	6
5	0.36	0.43	0.34	0.35	0.35	0.5	0.39	9
6	0.41	0.39	0.39	0.38	0.4	0.65	0.44	7
7	0.4	0.36	0.56	0.53	0.6	0.37	0.47	5
8	0.35	0.39	0.43	0.45	0.49	0.42	0.42	8
9	0.59	0.33	1	0.73	0.9	0.33	0.65	2

## Data Availability

The original contributions presented in this study are included in the article/Supplementary Material. Further inquiries can be directed to the corresponding author(s).

## Supplementary Materials

The following supporting information can be downloaded at: https://www.mdpi.com/article/10.3390/polym17070852/s1, Figure S1: (a) Tensile testing performed on the Instron Universal Testing Machine (b) Flexural testing performed on the Instron Universal Testing Machine (c) Pendulum testing machine. Table S1: Output responses of testing. Table S2: Analysis of variance for tensile strength. Table S3: Analysis of variance for flexural strength. Table S4: Analysis of variance for impact strength.
