# Supplementary material for: Optimization Scheme for 3D Printing of PLA–PHBV–PCL Biodegradable Blends for Use in Orthopedic Casting
Source: Polymers (Basel). 2025 Mar 22;17(7):852. doi: 10.3390/polym17070852 (PMC11991175; doi:10.3390/polym17070852)
Supplement: Supplementary file 1 [file polymers-17-00852-s001.zip › polymers-3505062-supplementary.pdf]

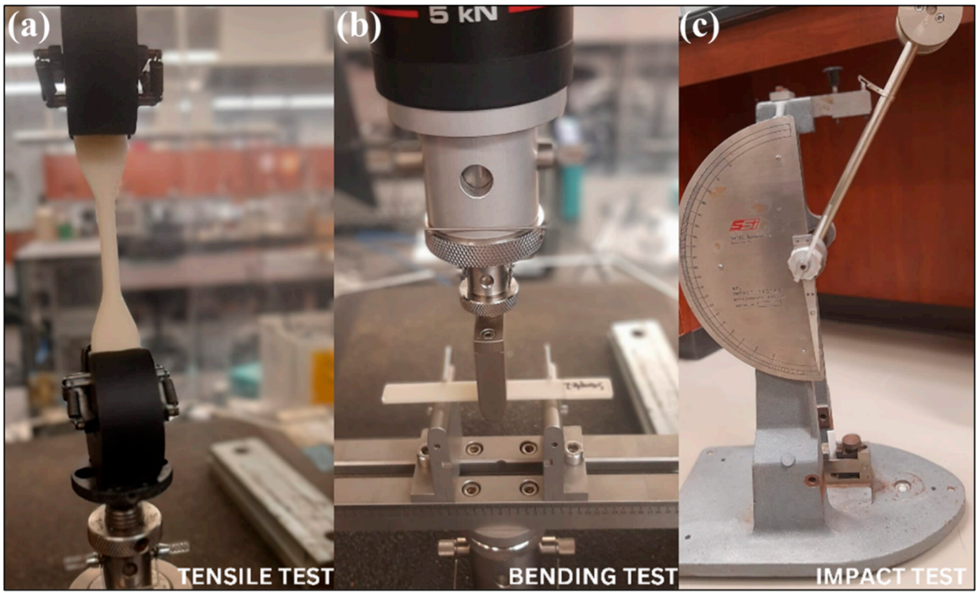

Figure S1: (a) Tensile testing performed on the Instron Universal Testing Machine (b) Flexural testing performed on the Instron Universal Testing Machine (c) Pendulum testing machine

Table S1: Output responses of testing

Test No.	Tensile Strength (MPa)	% Elongation at break	Young's Modulus (MPa)	Flexural Strength (MPa)	Flexural Modulus (MPa)	Impact Strength (J/m)
**1**	44.4 ± 4.4	68.5 ± 11.6	948.7 ± 25.1	54.6 ± 8.9	1549.3 ± 141	80.77 ± 5.6
**2**	29.5 ± 2.6	23.55 ± 4.3	638.5 ± 17.8	42.5 ± 2.5	1238.1 ± 63.11	96.17 ± 33
**3**	34.05 ± 7.14	70.8 ± 24.8	919.6 ± 153.4	32 ± 8.2	1093 ± 64.3	74.04 ± 20.5
**4**	20.72 ± 0.44	39 ± 1	519.34 ± 21.6	16.35 ± 0.92	504 ± 18.4	181.04 ± 14.8
**5**	22.9 ± 0.57	35.7 ± 6.5	544.2 ± 54.7	18.45 ± 0.071	595.8 ± 13.72	114.02 ± 10.7
**6**	27.16 ± 2.7	29.95 ± 3.9	634.4 ± 39.4	23.1 ± 0.283	767.7 ± 96.6	145.5 ± 25.6
**7**	26.6 ± 2.55	23.65 ± 1.3	830 ± 25	37.9 ± 0.64	1206 ± 7.8	66.53 ± 16.5
**8**	22.8 ± 0.2	28.6 ± 6.8	693.8 ± 10.7	31 ± 0.3	1015 ± 15.6	91.03 ± 12
**9**	36.1 ± 1.3	17.85 ± 1	1026.45 ± 32.3	47.4 ± 1.13	1489 ± 43.3	48.24 ± 5.73

Table S2: Analysis of variance for tensile strength

Source	DF	Seq SS	Contribution	Adj SS	Adj MS	F-Value	P-Value
Nozzle Temp.	2	88.11	18.92%	88.11	44.06	4.19	0.193
Raster Angle	2	233.59	50.18%	233.59	116.79	11.11	0.083
Layer Height	2	122.8	26.38%	122.8	61.4	5.84	0.146
Error	2	21.02	4.52%	21.02	10.51		
**Total:**	8	465.52	100%				

Table S3: Analysis of variance for flexural strength

Source	DF	Seq SS	Contribution	Adj SS	Adj MS	F-Value	P-Value
Nozzle Temp	2	48.58	3.53%	48.58	24.29	0.59	0.629
Raster Angle	2	960.43	69.81%	960.43	480.29	11.67	0.079
Layer Height	2	284.41	20.67%	284.41	142.21	3.46	0.224
Error	2	82.28	5.98%	41.14	41.14		
**Total:**	8	1375.71	100%				

Table S4: Analysis of variance for impact strength

Source	DF	Seq SS	Contribution	Adj SS	Adj MS	F-Value	P-Value
Nozzle Temp	2	613.5	4.45%	613.5	306.7	0.61	0.620
Raster Angle	2	10343.8	75.11%	10343.8	5171.9	10.32	0.088
Layer Height	2	1812.2	13.16%	1812.2	906.1	1.81	0.356
Error	2	1002.2	7.28%	1002.2	501.1		
**Total:**	8	13771.7	100%				
